# Further Aspects of Ochratoxin A-Cation Interactions: Complex Formation with Zinc Ions and a Novel Analytical Application of Ochratoxin A-Magnesium Interaction in the HPLC-FLD System

**DOI:** 10.3390/toxins6041295

**Published:** 2014-04-10

**Authors:** Miklós Poór, Mónika Kuzma, Gergely Matisz, Yin Li, Pál Perjési, Sándor Kunsági-Máté, Tamás Kőszegi

**Affiliations:** 1 Institute of Laboratory Medicine, University of Pécs, Pécs H-7624, Hungary; E-Mail: poor.miklos@pte.hu; 2 Department of Pharmaceutical Chemistry, University of Pécs, Pécs H-7624, Hungary; E-Mails: monika.kuzma@aok.pte.hu (M.K.); pal.perjesi@aok.pte.hu (P.P.); 3 Department of General and Physical Chemistry, University of Pécs, Pécs H-7624, Hungary; E-Mails: gmatisz@gamma.ttk.pte.hu (G.M.); liyincumt@gmail.com (Y.L.); kunsagi@gamma.ttk.pte.hu (S.K.-M.); 4 János Szentágothai Research Center, University of Pécs, Pécs H-7624, Hungary

**Keywords:** ochratoxin A, zinc ion, magnesium ion, fluorescence, HPLC-FLD

## Abstract

Ochratoxin A (OTA) is a mycotoxin produced by different *Aspergillus* and *Penicillium* species. Since its mechanism of action is not fully understood yet, it is important to gain further insight into different interactions of OTA at the molecular level. OTA is found worldwide in many foods and drinks. Moreover, it can also be detected in human and animal tissues and body fluids, as well. Therefore, the development of highly sensitive quantitative methods for the determination of OTA is of utmost importance. OTA most likely forms complexes with divalent cations, both in cells and body fluids. In the present study, the OTA-zinc interaction was investigated and compared to OTA-magnesium complex formation using fluorescence spectroscopy and molecular modeling. Our results show that zinc(II) ion forms a two-fold higher stable complex with OTA than magnesium(II) ion. In addition, based on the enhanced fluorescence emission of OTA in its magnesium-bound form, a novel RP-HPLC-fluorescence detector (FLD) method was also established. Our results highlight that the application of magnesium chloride in alkaline eluents results in an approximately two-fold increase in sensitivity using the HPLC-FLD technique.

## 1. Introduction

**Figure 1:**
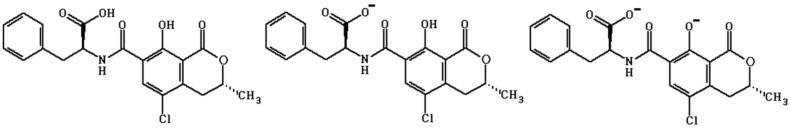
Chemical structures of nonionic, monoanionic and dianionic ochratoxin A (OTA).

The mycotoxin, ochratoxin A (OTA), is produced by several *Aspergillus* and *Penicillium* fungi [1,2]. OTA occurs worldwide in different foods and drinks, and because of its very high thermal stability, its eradication from the food chain at present seems to be impossible [3,4]. Its nephrotoxicity is only one of the toxic impacts attributed to OTA, and an increasing number of cellular/animal experiments and epidemiologic studies suggest carcinogenic and even other adverse effects of it [5,6,7]. The toxin is built up from a dihydroisocoumarin moiety linked to L-phenylalanine (Figure 1). OTA binds to human serum albumin (HSA) with a very high affinity [8]. Therefore, its plasma half-life is about one month in humans, resulting in long-term toxicity [9,10]. Molecular interactions of OTA seem to be a very complex area to understand, because OTA forms complexes not only with proteins, but also with ions, which might have considerable importance in biological systems [11,12,13]. For example, it is possible that the OTA-Fe^3+^ complex is partly responsible for the OTA-mediated increased reactive oxygen species (ROS) production [11,12]. In addition, as it was demonstrated in our previous work, dianionic OTA forms complexes with both alkali and alkaline earth metal ions [13]. The most remarkable interaction was detected in the case of magnesium(II) ion, which gives by far the highest stable complex among the three studied alkaline earth metal ions (Mg^2+^, Ca^2+^ and Ba^2+^) [13].

Zinc is an essential trace element that is incorporated into a number of human proteins, e.g., metallothioneins or superoxide dismutase (SOD) [14]. Zinc is required for a wide range of physiological functions: it plays an important role in nucleic acid metabolism, cell replication and signaling, tissue repair and growth, *etc.* [15,16]. The involvement of zinc ions in the cellular defense against OTA has been published recently [17]. Zinc supplementation of OTA-exposed cells results in the reduction of reactive oxygen species (ROS) levels and oxidative DNA damage. Furthermore, it increases SOD activity [18].

There is a wide range of analytical methods suitable for the sensitive quantification of OTA. Primarily, high performance liquid chromatographic (HPLC) methods are the most commonly used processes for food and other biological samples [19,20,21]. Since OTA is a highly fluorescent molecule, its quantitative determination with a fluorescence detector (FLD) can be achieved at a comparable sensitivity to mass spectrometry (MS). In an aqueous environment, OTA is present, depending on the pH and on the microenvironment, in three different forms: nonionic, monoanionic (OTA^−^) and dianionic (OTA^2−^), respectively (Figure 1) [13,22]. The nonionic and monoanionic forms of OTA show the same fluorescence spectral properties (λ_exc_ = 334 nm; λ_em_ = 451 nm) and intensities. Nevertheless, OTA^2−^ has a much higher fluorescence signal with an excitation and emission wavelength maxima at 380 and 443 nm, respectively [8,22]. In many cases, acidic eluents (at about pH 2.5–3.0) are used to determine the concentration of OTA by HPLC-FLD [19,23,24]. Since pKa values of ochratoxin A are within 4.3–4.4 (carboxyl group) and 7.0–7.3 (phenolic hydroxyl group) [5], under these conditions, OTA is present predominantly in nonionic form. Further studies highlighted that using alkaline eluents, the sensitivity of OTA detection significantly increases in thin layer chromatography (TLC) and HPLC-FLD applications [25,26,27]. This phenomenon is not surprising, since OTA^2−^ provides much higher fluorescence emission intensity than the other two forms [8,22]. At pH 9.0 or at higher pH values, OTA is present almost completely in dianionic form; therefore, alkaline eluents are applied between pH 9.0 and 10.0 [25,26,27]. Moreover, OTA exerts about a two-fold higher fluorescence signal in the magnesium bound form (λ_exc_ = 375 nm; λ_em_ = 427 nm) than unbound OTA^2−^ (λ_exc_ = 380 nm; λ_em_ = 443 nm) [13]; therefore, we hypothesized that the application of the Mg^2+^-OTA^2−^ interaction might be a suitable tool to develop a novel, more sensitive chromatographic technique than those published in the literature.

To our knowledge, in our study, we were the first to examine the direct interaction between OTA and zinc ions. The stability constant of the Zn^2+^-OTA^2−^ complex and the thermodynamics of the complex formation were investigated at physiological pH (7.4) using fluorescence spectroscopy. Then, the newly discovered interaction was compared to the parameters of the previously described Mg^2+^-OTA^2−^ complex. In order to explain the differences between the complex stabilities, molecular modeling studies were also performed. Our second major goal was the analytical utilization of the Mg^2+^-OTA^2−^ interaction in an HPLC-FLD system. A novel and highly sensitive chromatographic method was developed based on the enhanced fluorescence of the Mg^2+^-OTA^2−^ complex. Our application allows us to detect OTA with an approximately two-fold higher sensitivity on an HPLC-FLD system without considerable elevation in the costs of the method.

## 2. Results and Discussion

### 2.1. Interaction of OTA with Zinc and Magnesium Ions: Complex Stability and Thermodynamics

**Figure 2:**
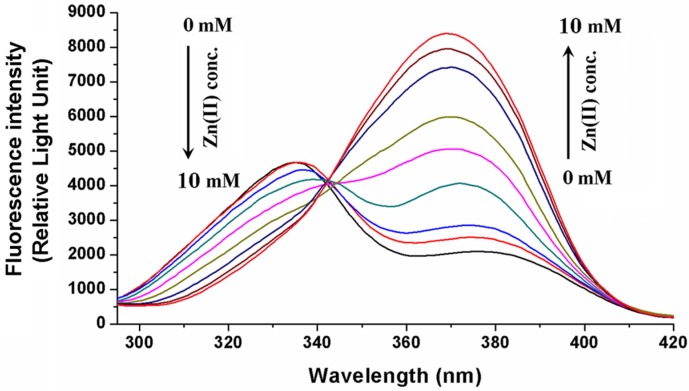
The fluorescence excitation spectra of 1 µM OTA (λ_em_ = 380 nm) in the presence of increasing amounts of Zn^2+^ in ammonium acetate buffer (pH 6.4).

**Figure 3:**
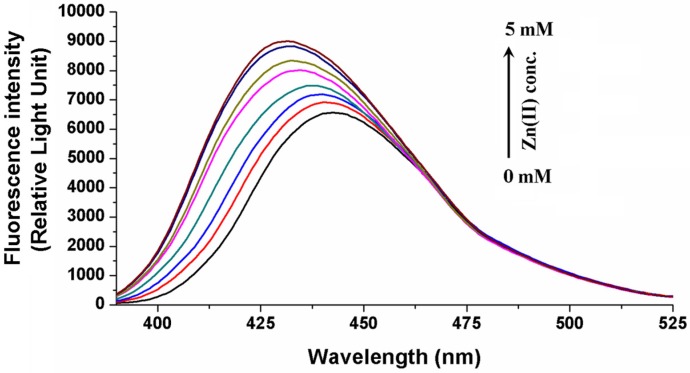
The fluorescence emission spectra of 1 µM OTA (λ_exc_ = 380 nm) in the presence of increasing Zn^2+^ concentrations in TRIS-HCl buffer (pH 7.4).

Similarly to magnesium ions [13], zinc ions also form a complex with OTA and show a strong preference toward the dianionic form of OTA. A major decrease with a consecutive increase of the peak intensities at 332 nm and 370 nm was observed in the excitation spectra of OTA upon the addition of Zn^2+^ in a 0.03 M ammonium acetate buffer at pH 6.4 (Figure 2). These variations are due to a decrease in the amount of monoanionic OTA with an increase in the amount of the Zn^2+^-OTA^2−^ complex in the solution, respectively. The complex formation with zinc ions results in a blue shift (443 nm→431 nm) of the emission peak of OTA^2−^. Furthermore, its fluorescence intensity significantly elevates (Figure 3). Zn^2+^-OTA^2−^ complexation causes a less pronounced increase in fluorescence intensity compared to that of its Mg^2+^ complex (in TRIS-HCl buffer, pH 7.4). Nevertheless, the stability constant of the Zn^2+^-OTA^2−^ complex is 0.38 log units higher than that of Mg^2+^-OTA^2−^. The determined log*K* values of cation-OTA^2−^ complexes at 25 °C and the corresponding thermodynamic parameters are summarized in Table 1. The thermodynamic parameters show that the increases of entropy at the complex formations are important in each case. The removal of the ions from their solvation shells results in entropy production, which might be one of the driving forces in the complex formation. These assumptions further support our previous argument that the dehydration of magnesium ions possesses a high importance during the process [13]. In addition, presumably, the partial dehydration of OTA also plays a role in the entropy production. It should be noted that the binding constant of Mg^2+^-OTA^2−^ in TRIS-HCl (pH 7.4) is also 0.4 log units higher than that in phosphate buffered saline (PBS) buffer (pH 7.4). This discrepancy could result from the fact that PBS buffer contains a very high concentration of sodium ions (almost 150 mM), which can also form complexes with OTA. Even though the stability of Na^+^-OTA^2−^ [13] is much lower than that of Mg^2+^-OTA^2−^, a large amount of sodium ions is still able to compete with Mg^2+^ for binding with OTA^2−^.

**Table 1:** Log*K* values and thermodynamic parameters of Zn^2+^-OTA^2−^ and Mg^2+^-OTA^2−^ complexes in TRIS-HCl (pH 7.4) and phosphate buffered saline (PBS) (pH 7.4) buffers.

Complex	Log *K* (25 °C)	Δ*H* (kJ·mol^−1^)	Δ*S* (J·mol^−1^·K^−1^)	Δ*G* (kJ∙mol^−1^; 25 °C)
Zn^2+^-OTA^2−^ (TRIS-HCl)	3.78 ± 0.01	−10.4 ± 2.6	34.0 ± 9.8	−21.6 ±0.1
Mg^2+^-OTA^2−^ (TRIS-HCl)	3.40 ± 0.01	−8.3 ± 0.4	37.6 ± 1.5	−19.4 ± 0.1
Mg^2+^-OTA^2−^ (PBS)	3.00 ± 0.01	−3.0 ± 1.4	47.1 ± 4.5	−17.1 ±0.1

Depending on cell type, the intracellular zinc concentration could be considerably different; however, the free fraction of Zn^2+^ is always very low [28,29,30]. Previous studies highlighted that both a sharp increase and also a decrease of intracellular free zinc levels are unfavorable in living cells [31,32]. However, in living cells, OTA can interact with different proteins and even other substances. The stability of the Zn^2+^-OTA^2−^ complex suggests that the complex formation probably occurs even *in vivo*. Based on our investigation and the previously published studies with OTA and zinc [17,18], it is possible that the direct Zn^2+^-OTA^2−^ interaction may play a role in the zinc-dependent negative effects of OTA.

### 2.2. Molecular Modeling Studies

**Figure 4:**
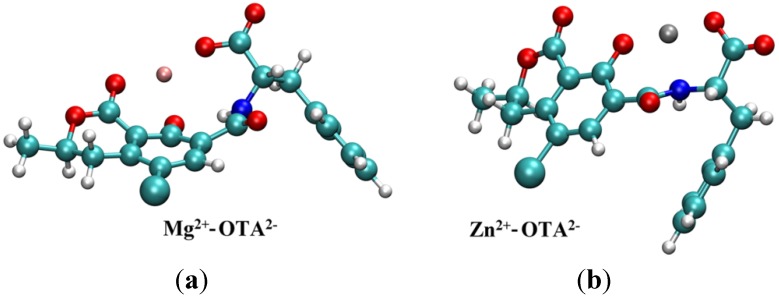
(**a**) [Mg^2+^-OTA^2−^] structure. The Mg^2+^ ion primarily interacts with three partially negatively charged oxygen atoms. Based on NBO analysis as implemented in the Gaussian program, the total charge on Mg^2+^ is 1.65 e, while there are −0.99 e, −0.87 e and −0.78 e charges on the three oxygen atoms (one in the carboxyl group, the phenolic oxygen and the oxo group). The amide nitrogen possesses a −0.63 e charge compared to the −0.64 e charge in the free OTA^2−^; (**b**) [Zn^2+^-OTA^2−^] structure. The Zn^2+^ ion can be found close to the two partially negatively charged oxygen atoms and to the nitrogen atom of the amide group. The total charge on Zn^2+^ is 1.38 e, while there are −0.83 e and −0.81 e charges on the two oxygen atoms (carboxyl and phenolic, respectively) and −0.73 e on the nitrogen atom.

The structures of cation-OTA complexes are detailed in Figure 4. The ΔH and ΔS thermodynamic functions have been calculated. The formation of the Zn^2+^-OTA^2−^ complex is preferred energetically by 124.2 kJ/mol compared to the formation of the same Mg^2+^ complex. After temperature corrections, the difference between the obtained ΔH_f_ values was 122.4 kJ/mol. Thus, the results of the calculation show that the formation of the Mg^2+^-OTA^2−^ complex is less favored enthalpically in comparison to the formation of the Zn^2+^-OTA^2−^ complex. The above difference, compared to the experimental values, might be raised by the limited consideration of the solvation shell of the ions in the theoretical model.

By comparing the entropy changes, the formation of the Mg^2+^-OTA^2−^ complex is preferred by 8–11 J∙mol^−1^K^−1^, depending on the applied density functional method (M06-2X and B3LYP, respectively). Based on the calculations, the difference in the ΔS of the two processes (the complex formation of Mg^2+^ and Zn^2+^ according to Equations (2) and (3)) is derived almost completely from the vibrational entropy contributions (S_vib_). The change of S_vib_ is associated mainly with the formation of water clusters from the water solvation shell of the cations, *i.e*., to the partial decomposition of the solvation shell of the Mg^2+^ and the Zn^2+^ ions.

### 2.3. Influence of pH on the Mg^2+^-OTA^2−^ Complex Formation

**Figure 5:**
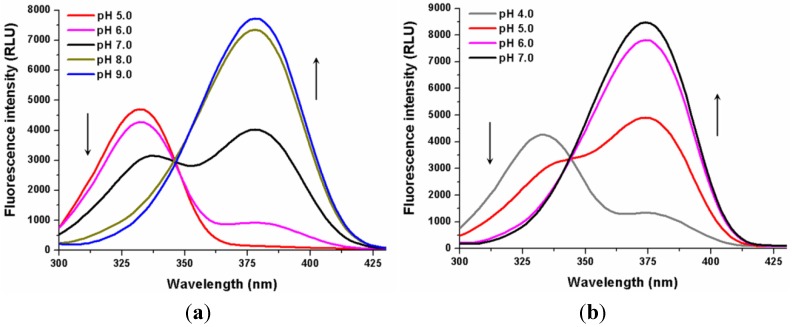
Fluorescence excitation spectra of 1 µM OTA in the absence (**a**) (λ_em_ = 443 nm) and in the presence (**b**) (λ_em_ = 427 nm) of 50 mM MgCl_2_ in 0.03-M ammonium acetate buffers at increasing pH (RLU = Relative Light Unit).

Before chromatographic measurements, the influence of pH on the Mg^2+^-OTA^2−^ complex formation was studied in 0.03-M ammonium acetate buffers (pH 4.0–9.0). Figure 5 shows the fluorescence excitation spectra of OTA (1 µM) alone and in the presence of 50 mM Mg^2+^ at different pH values. In the absence of magnesium ions, dianionic OTA appears only at pH 6.0, and tuning to pH 9.0 is needed to achieve the maximal amount of OTA^2−^. On the other hand, Mg^2+^-OTA^2−^ complexes were detected even at pH 4.0, in addition to at pH 7.0, the total amount of OTA was present in the magnesium-bound form. These results demonstrate that the Mg^2+^-OTA^2−^ complex is able to form within wide pH ranges.

### 2.4. Analytical Application of the Mg^2+^-OTA^2−^ Interaction on HPLC-FLD

**Figure 6:**
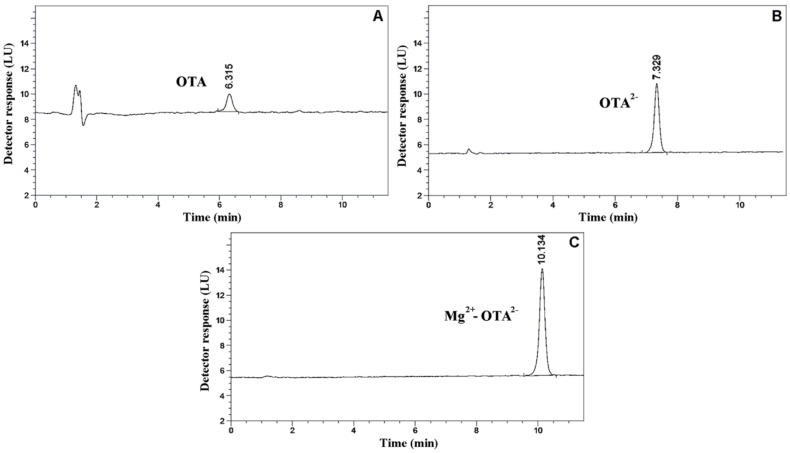
Chromatograms of OTA solutions (in ethanol, 25 nM) under different elution conditions. (**A**) Using the acidic mobile phase; (**B**) using the alkaline mobile phase; (**C**) using the alkaline mobile phase with magnesium ions (LU = Light Unit).

Our newly developed RP-HPLC-FLD method (“eluent C”) was linear in the range of 2.5–1000 nM for OTA (*R*^2^ = 0.9995). Data for precision (both intra-day and inter-day data) were within five RSD%. The limit of detection (LOD) and the limit of quantification (LOQ) values were found to be 1 nM and 2.5 nM, respectively. It is important to note that in our study, the major focus was on the comparison of the analytical performance of the three described eluents. Evidently, by the elevation of the injection volume or by applying a more sensitive fluorescent detector, the sensitivity of the method can be substantially higher. Figure 6 demonstrates the chromatograms of 25 nM OTA using acidic eluent (“Eluent A”), alkaline eluent (“Eluent B”) and alkaline eluent containing MgCl_2_ (“Eluent C”). It is clearly seen that there are major differences between the sensitivities of the three methods. The determined LOD and LOQ values were 10 nM and 20 nM in the case of “Eluent A”, as well as 2 nM and 5 nM in the case of “Eluent B”, respectively. Our findings confirm that the application of alkaline mobile phase (compared to the acidic mobile phase) in HPLC determination results in a substantial increase of the fluorescence of OTA. These results are in good agreement with the data of the previously published studies [26,27]. On the other hand, our experiments also demonstrate that the admixture of magnesium ions into the alkaline mobile phase causes a further increase in fluorescence. Our data prove that there is a two-fold increase in LOD and LOQ when magnesium ions are used in the alkaline mobile phase. This observation highlights that the OTA-magnesium interaction is suitable for achieving a substantially more sensitive determination of OTA applying an RP-HPLC-FLD system without the considerable elevation of costs or using expensive sample preparation. Based on these results we suggest the application of magnesium supplemented alkaline eluents in the analytical determination of OTA using the HPLC-FLD technique.

## 3. Experimental Section

### 3.1. Reagents

Ochratoxin A (OTA) was purchased from Sigma-Aldrich; 5,000 µM stock solution (in ethanol, Reanal, spectroscopic grade) was prepared and kept at 4 °C, protected from light. Phosphate buffered saline (PBS: 137 mM NaCl, 2.7 mM KCl, 3.6 mM NaH_2_PO_4_, 1.5 mM K_2_HPO_4_ in tridistilled water; pH 7.4), 0.1 M TRIS-HCl buffer (pH 7.4) and 0.03 M ammonium acetate buffers (pH 4.0, 5.0, 6.0, 7.0, 8.0 and 9.0) were used for spectroscopic measurements. Triethylamine (99%, purchased from Sigma-Aldrich), acetonitrile (gradient grade, obtained from Carlo Erba) and methanol (optigrade, LGC Promochem) were used for chromatographic measurements.

### 3.2. Fluorescence Spectroscopic Investigations

A Hitachi F-4500 fluorescence spectrophotometer and a Fluorolog τ3 spectrofluorimetric system were applied for the investigation of the Zn^2+^-OTA^2−^ and Mg^2+^-OTA^2−^ complex formation. Excitation and emission spectra were recorded at 25 °C in the presence of air.

First to examine the potential preferences of zinc ion towards monoanionic or dianionic forms of OTA, increasing ZnCl_2_ concentrations (50 µM–30 mM) were added to 1 µM OTA in 0.03 M ammonium acetate buffer (pH 6.4). Under these conditions both OTA^−^ and OTA^2−^ are present in the solution, and OTA^−^ predominates.

For the determination of binding constants and thermodynamic parameters at physiological pH, increasing ZnCl_2_ and MgCl_2_ concentrations (50 µM–50 mM) were added to 1 µM OTA in TRIS-HCl buffer (pH 7.4). Only the Mg^2+^-OTA^2−^ interaction was tested in PBS (pH 7.4), because zinc precipitates under these circumstances. Fluorescence emission spectra were recorded using 380 nm as the excitation wavelength. Measurements were performed at 20, 25, 30, 35 and 40 °C, respectively. Intensities obtained at 432 nm and 425 nm emission wavelengths were used for the binding constant evaluation of Mg^2+^-OTA^2−^ and Mg^2+^-OTA^2−^, respectively. Binding constants (*K*, expressed in dm^3^/mol) were determined applying the previously described procedure [13,33]. Accordingly, binding constants were calculated from emission intensities by the Hyperquad2006 program package [34] using 1:1 stoichiometry and applying a non-linear least-squares fit:

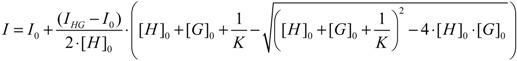
(1)
where *I* denotes the fluorescence emission intensity of OTA in the presence of cation (Zn^2+^ or Mg^2+^); *I_0_* denotes the fluorescence emission intensity of OTA in the absence of cation; *I_HG_* denotes the fluorescence emission intensity of pure cation-OTA^2−^ complex, which is calculated by Hyperquard2006, *K* denotes the binding constant and [*H*]_0_ and [*G*]_0_ are the initial concentration of OTA and cation, respectively. Thermodynamic parameters were determined using the van’t Hoff theory.

Before the chromatographic measurements, the effect of pH on Mg^2+^-OTA^2−^ complex formation was also investigated. One-micromolar OTA and 50 mM MgCl_2_ were applied in 0.03-M ammonium acetate buffer at pH 4.0, 5.0, 6.0, 7.0, 8.0 and 9.0, respectively. Emission spectra were recorded using 380 nm (in the absence of Mg^2+^) and 375 nm (in the presence of Mg^2+^) as the excitation wavelengths.

### 3.3. Molecular Modeling of Zn^2+^-OTA^2−^ and Mg^2+^-OTA^2−^ Complexes

Theoretical calculations have been carried out to compare the interaction of Zn^2+^ and Mg^2+^ ions with the OTA^2−^ dianion. The first tightly bound solvation shell of the ions were considered to be composed by six octahedrally coordinated water molecules [35,36]. The lowest energy levels of the cation-OTA^2−^ complexes were searched on the basis of initial trial structures published earlier for similar systems [13]. The geometries were optimized at the B3LYP-D3 theoretical level using the TeraChem 1.5K program [37,38,39,40]. The 6-311++G(2d,2p) basis set [41,42] has been applied until Mg^2+^ in the periodic table, and the Ahlrichs TZV basis set has been used [41,42,43] in the case of Zn^2+^. After the geometry optimizations, the energies of the ions and compounds were calculated by the processes written below (Equations (2) and (3)) at the MP2(full)/aug-cc-pVTZ theoretical level and the basis set as implemented in the Gaussian 09 program. The thermochemical analysis has been performed on the M06-2X/6-311++G(2d,2p) [44] geometries by using the same G09 program.

[Mg^2+^(H_2_O)_6_] + OTA^2−^↔[Mg^2+^OTA^2−^] + (H_2_O)_6_ (2)

[Zn^2+^(H_2_O)_6_] + OTA^2−^↔[Zn^2+^OTA^2−^] + (H_2_O)_6_ (3)

### 3.4. Chromatographic Conditions on HPLC-FLD System

Working solutions of OTA standards were prepared by dilution of the 5000 μM stock solution with ethanol to give a concentration range of 2.5 to 1000 nM (2.5; 5; 10; 50; 100; 500; 1000 nM, respectively). The working standard solutions were stored at 4 °C, preserving their stability for at least 14 days.

The integrated high performance liquid chromatography system (Agilent 1100), being qualified and validated according to pharmaceutical requirements, was equipped with a quaternary pump, a degasser, an autosampler, an injector with a 100-μL sample loop, a column oven and a fluorescent detector. Data were recorded and evaluated using Agilent ChemStation (Rev.B.03.02-SR2) software. Separation was performed on a LiChroCART^®^ 4 mm × 125 mm, 5-µm particle size, Merck Purospher STAR^®^ RP-18e (endcapped) column with a guard cartridge (TR-C-160-K1; ABLE&E-Jasco) using the binary gradient mobile phase.

Since, in numerous cases, acidic eluents are used for the quantitative analyses of OTA by HPLC-FLD, applying the identical chromatographic system, a comparative analysis was performed with a previously studied acidic eluent (acetonitrile/CH_3_COONa buffer, 5 mM, pH 3.0/methanol; 30/40/30, v/v) to investigate the effect of pH on fluorescence detection under chromatographic conditions. Our choice of the above described acidic eluent was based on the review article of Afsah-Hejri and Jinap [19] (where more than 10 different eluents were tested), who evaluated it as the most sensitive method among the acidic eluents. The acidic mobile phase will be referred to as “Eluent A” in the following.

Our applied alkaline mobile phase consisted of triethylamine buffer (triethylamine hydrochloride/triethylamine 5 mM, pH 9.8) and acetonitrile. The gradient elution was as follows: from 0 to 10 min, ACN was increased from 17.5% (v/v) to 25%, while triethylamine buffer was decreased from 82.5% to 75%. From 10 to 12 min, ACN and buffer returned to the initial values. This alkaline mobile phase will be referred to as “Eluent B” in the following. In the case of the third eluent, we applied the same buffers and conditions as for “Eluent B” with the exception that 5 mM MgCl_2_ was dissolved in the triethylamine buffer. This solution will be referred to as “Eluent C” in the following. All eluents were filtered through a glass filter (porosity of 4) before use. Chromatography was performed at 30 °C, and the flow rate was 0.85 mL/min. The peak areas were monitored by a fluorescence detector (λ_exc_ = 386 nm and λ_em_ = 440 nm, PMT gain = 15, attenuation = 50), the injection volume was 5 μL. Under acidic conditions (“Eluent A”) all settings were the same except the excitation and emission wavelengths: λ_exc_ = 333 nm and λ_em_ = 460 nm, respectively.

## 4. Conclusions

The Zn^2+^-OTA^2−^ and Mg^2+^-OTA^2−^ interaction was examined using steady-state fluorescence spectroscopy and molecular modeling. Our results show that zinc(II) ions form a stable complex with OTA and show preference toward its dianionic form. The stability of the Zn^2+^-OTA^2−^ complex is 2.4-fold higher than that of Mg^2+^-OTA^2−^. The thermodynamic parameters determined from van't Hoff plots show that these complex formations are favored by both enthalpic and entropic contributions. At elevated temperatures, the higher preference toward Zn^2+^ ions is rather due to enthalpic contribution. The entropy increase in the two discussed complex formations could be attributed to the partial desolvation of the metal ions. In addition, the analytical application of the Mg^2+^-OTA^2−^ complex formation was also tested in an RP-HPLC-FLD system. We demonstrated that the supplementation of alkaline eluent with magnesium chloride is a cheap and simple process, being suitable for achieving a two-fold higher sensitivity compared to the simple alkaline eluent.
